# Supplementary material for: Prediction of edapho-climatic parameters in the incidence of Campylobacter spp. in northwestern Mexico
Source: PLOS Glob Public Health. 2024 Apr 25;4(4):e0002812. doi: 10.1371/journal.pgph.0002812 (PMC11045143; doi:10.1371/journal.pgph.0002812)
Supplement: Attachment — Submitted filename: PGPH-D-23-01841_Review.pdf [file pgph.0002812.s002.pdf]

Summary: The manuscript describes an interesting approach of using mathematical modelling of geographic, environmental and geological (edaphic) factors to confirm and predict the presence of *Campylobacter* in the environment. They hope to improve surveillance by linking certain edaphic factors to higher incidence of human Campylobacteriosis.

Abstract

- The Abstract adequately summarises the study.

Introduction

- The Introduction gives a detailed background to the study, some sections are lengthy and would benefit from shortening, some sentences need rewriting (see details below).
- The aim is clearly stated.

Line 30	from not form
Line 53/54	*Campylobacter* is the primary bacterial agent, as far as I am aware there are viral causes that are more prevalent such as Rota- and Norovirus.
Line 54	I suggest “primary etiological agent” not first etiological agent
Line 55	The authors talk about WHO but reference the FDA, why not reference WHO?
Line 57	if children had diarrhoea then they weren’t asymptomatic?
Line 59/61	*Campylobacter jejuni* not jenuni
Line 60	“…in 40% of cases”, does this refer to Mexico or globally, please specify
Line 61	Does reference 7 belong to Line 58?
Line 70/71	suggested rewriting this sentence:” Soil, manure, aquatic environments and water are the natural niches where *Campylobacter spp*. are found and transmission to humans and susceptible animals can occur.” Or similar
Line 73/74	suggested rewrite:“The growth and survival of *Campylobacter* spp is related to external environmental factors such as water, heat, UV radiation and salinity.”
Line 85/98	*Campylobacter* in italics

Methods

- Over what time period where the various data points collected?

- The authors give a lot of detail about the kriging method, some of which might be better suited for the introduction or the discussion in conjunction with their findings.
- However, they do not explain the BIO designations used in the Figures and these are also not mentioned anywhere in the results.

Line 142 some information on the 19 variables that make up the profile would be interesting here.

Results

- The authors describe BIO variables in the Figure legends but this is not described the in the result text and it is difficult to see how they relate to the tables, could an extra column be introduced in Table 2 for example, or am I misunderstanding something?
- I do not feel that the Figure titles and legends give me enough information to understand the figures completely.

Line 201 References to ANUCLIM and Worldclim is needed here, particularly as the authors refer to them for the meaning of the colours in Figures 2-6, which are not given in a legend.

Line 214 *Campylobacter spp*., incidence

Discussion

- I struggle to understand how the authors put their findings into context with the literature. My expectations from the Introduction were that the model would describe the interplay of several factors to create ideal conditions for *Campylobacter* to be found but the authors seem to discuss individual factors such as humidity or temperature. I am obviously not an expert on this but I feel that more could have been made of the data?

- Overall, the lack of *Campylobacter spp*. data is mentioned several times, would a lack of data or differences in reporting between the areas skew the results of the model? If so, this needs to be discussed in more detail.

- The conclusions section is not a conclusion but more a discussion of limitations, I would suggest discussing the limitations and expand on the conclusions described in the Abstract at the end of the discussion section.

Line 220 *Campylobacter* is a facultative anaerobe, so I think this needs rephrasing for accuracy.

Line 222 the authors state that “recent studies have shown…” yet one of the papers referenced is from 2007

Line 223 Viable but non culturable state

Line 224-226 This sentence is hard to understand, do the authors mean maintenance instead of permanence for example? Please rephrase.

Line 236 *C. jejuni*

Line 238 the municipality

Line 251 Rollins *et al* is the original reference for the 120 days, not the papers sited, other papers show a shorter duration so this should be acknowledged.

Line 323/324 “*Campylobacter spp*. are the second most frequently reported cause of foodborne illness.” Where? In Mexico or globally? Also this is not the main conclusion of this data, see comment above.
