# Supplementary material for: Prediction of edapho-climatic parameters in the incidence of Campylobacter spp. in northwestern Mexico
Source: PLOS Glob Public Health. 2024 Apr 25;4(4):e0002812. doi: 10.1371/journal.pgph.0002812 (PMC11045143; doi:10.1371/journal.pgph.0002812)
Supplement: Attachment — Submitted filename: Rebuttal_letter_Chaves_etal.pdf [file pgph.0002812.s003.pdf]

Dear editor, dear reviewers:

Please find below the response to the reviewers' comments to our manuscript with the revised title “Prediction of edapho-climatic parameters in the incidence of *Campylobacter* spp. in northwestern Mexico”, please find the comments in blue.

General comment:

- We will make all necessary changes to facilitate the comprehension of the results and their relevance; this will include review the data in the figures and in the text, and improve the thread of thought between the results obtained and their relevance and implications.

Reviewer # 1

This manuscript is describing the link between environment and campylobacter spp, using a prediction model. I am a little confused how given you state that there is limited records and surveillance of Campylobacter in Mexico, there is no animal surveillance and so I am assuming your predictions are based on human cases, which of course not all will be reported and so I am concerned over the small number of records used within the model and so the links to Campylobacter may be not as concrete as the authors allude to. I question whether all of the figures are needed and if perhaps they could be reduced?

Minor comments:

L56 Is there any animal surveillance? Is campylobacter looked for in animal populations, even for research studies? Obviously, most environmental Campylobacter will be attributed to an animal, what species of campylobacter are found in humans in Mexico?

Information included:

In Mexico, reports of campylobacteriosis in animals are scarce and the public health impact is unknown. Isolated investigations have reported the presence of *Campylobacter* spp. in humans, food, and food-animals [4]. Species reported include *Campylobacter jejuni*, *Campylobacter coli* and *Campylobacter fetus* [5, 6, 7].

L85 Reference is needed for soil survival, I would expect some further details on how long Campylobacter spp. have been reported to survive in soil.

Information included:

Soil, manure, aquatic environments and water are the natural niches where *Campylobacter* spp. are found and transmission to humans and susceptible animals can occur [16]. The growth and

survival of *Campylobacter* spp is related to external environmental factors such as water, heat, UV radiation and salinity [17].

L153 Campylobacter spp. should be in italics

Corrected.

Results: Campylobacter data from the surveillance schemes, did they all use the same standardise culture methods/detection methods?

Data on the presence of Campylobacter spp. were taken into account without differentiating the diagnostic tests from studies that included the variables we are evaluating: edaphic factors, presence of water bodies, vegetation, land use and meteorological factors.

L269 What literature review? Do you mean you performed a review of the literature?

Information included in methodology:

A systematic search of world scientific literature was carried out in which edaphic, meteorological, water bodies, vegetation and land use factors associated with the presence of *Campylobacter* spp. in soil were established. At the same time, epidemiological records of *Campylobacter* spp. were consulted, without success, from Mexican public health and agricultural authorities and epidemiological bulletins. According to what was consulted in these institutions and corroborated by the systematic search carried out, it was not possible to obtain data for the northwestern region of Mexico that would be useful for our study.

Reviewer # 2

Summary: The manuscript describes an interesting approach of using mathematical modelling of geographic, environmental and geological (edaphic) factors to confirm and predict the presence of Campylobacter in the environment. They hope to improve surveillance by linking certain edaphic factors to higher incidence of human Campylobacteriosis.

Abstract

• The Abstract adequately summarises the study.

Thank you.

Introduction

Line 30 from not form

Corrected.

Line 53/54 Campylobacter is the primary bacterial agent, as far as I am aware there are viral causes that are more prevalent such as Rota- and Norovirus.

Corrected:

Campylobacter spp. -curved Gram-negative bacillus-, is a leading cause of foodborne bacterial zoonotic infections worldwide [1].

Line 54 I suggest "primary etiological agent" not first etiological agent

Corrected.

Line 55 The authors talk about WHO but reference the FDA, why not reference WHO?

Corrected.

Line 57 if children had diarrhea, then they weren't asymptomatic?

Clarified:

Campylobacter spp. has been found in asymptomatic children and in children with diarrhea [4,8].

Line 59/61 Campylobacter jejuni not jenuni

Corrected.

Line 60 "…in 40% of cases", does this refer to Mexico or globally, please specify

Clarified:

In addition, in Mexico Guillain-Barré syndrome is the most frequent cause of paralysis in children under 15 years of age and has been correlated with previous *C. jejuni* infection in up to 40% of cases [8].

Line 61 Does reference 7 belong to Line 58?

Corrected.

Line 70/71 suggested rewriting this sentence:" Soil, manure, aquatic environments and water are the natural niches where Campylobacter spp. are found and transmission to humans and susceptible animals can occur." Or similar

Corrected.

Line 73/74 suggested rewrite:"The growth and survival of Campylobacter spp is related to external environmental factors such as water, heat, UV radiation and salinity."

Corrected.

Line 85/98 Campylobacter in italics

Corrected.

Methods

• Over what time period where the various data points collected?

Included:

Data corresponding to edaphic variables were obtained from 170 samples of the soil profile dataset at scale 1:250 000, series II (National Continuous). Serie II: 2002 - 2007 [27]:…

• The authors give a lot of detail about the kriging method, some of which might be better suited for the introduction or the discussion in conjunction with their findings.
• However, they do not explain the BIO designations used in the Figures and these are also not mentioned anywhere in the results.

Included in the figures and results.

Line 142 some information on the 19 variables that make up the profile would be interesting here.

Included.

Table 1. Climatic data obtained from 23 meteorological stations present in the three municipalities and adjacent ones from the National Meteorological Service and the corresponding calculations made for the 19 bioclimatic profiles in Mexicali (Baja California), Santa Cruz (Sonora) and Janos (Chihuahua), Mexico.

Variable	Mexicali, Baja California	Santa Cruz, Sonora	Janos, Chihuahua
BIO1 Average annual temperature	24.4 – 15.1	17.7 – 16.7	26.1 – 25.6
BIO2 Mean diurnal temperature range (temp max - temp min; monthly average)	20.5 – 12.4	18.6 – 18.0	17.9 – 16.8
BIO 3 Isothermality (BIO1/BIO7)*100	56.0 – 45.4	53.8 – 53.2	51.9 – 48.0
BIO4 Seasonality of temperature (coefficient of variation)	783.5 – 142.8	6.42 – 6.31	628.7 – 51.0
BIO5 Maximum temperature of the warmest month	43.8 – 27.0	36.8 – 36.1	34.2 – 33.6
BIO6 Minimum temperature of the coldest month	8.4 – 1.3	1.55 – 1.01	0.0 – -1.0
BIO7 Annual temperature range (BIO5-BIO6)	39.9 – 19.7	34.6 – 33.7	34.3 – 33.83
BIO8 Average temperature of the wettest quarter	33.6 – 21.8	24.8 – 23.6	23.9 – 22.5
BIO9 Average temperature of the driest quarter	18.6 – 13.5	13.7 – 13.0	14.0 – 13.5
BIO10 Average temperature of the warmest quarter	27.3 – 15.7	21.6 – 20.8	21.0 – 20.78
BIO11 Average temperature of the coldest quarter	14.5 – 12.0	10.7 – 9.4	21.0 – 20.78
BIO12 Annual precipitation	271.6 – 32.5	513.7 – 503.4	467.1 – 463.0
BIO13 Precipitation of the rainiest month	64.5 – 6.7	127.6 – 115.9	113.8 – 103.1
BIO14 Precipitation of the driest month	1.4 – 0.0	8.4 – 5.8	8.2 – 6.8
BIO15 Seasonality of precipitation (coefficient of variation)	115.4 – 11.0	7.64 – 7.40	94.4 – 85.5
BIO16 Precipitation of the rainiest quarter	77.9 – 6.9	288.2 – 283.4	278.4 – 258.6
BIO17 Precipitation of the driest quarter	130.3 – 4.7	74.3 – 52.8	33.8 – 27.1

BIO18 Precipitation of the hottest quarter	23.5 – 0.1	36.1 – 31.0	65.6 – 56.7
BIO19 Precipitation of the coldest quarter	146.2 – 7.7	109.9 – 83.5	73.9 – 42.9

Results

• The authors describe BIO variables in the Figure legends but this is not described the in the result text and it is difficult to see how they relate to the tables, could an extra column be introduced in Table 2 for example, or am I misunderstanding something?

Information included:

The high incidence sites for Janos (CHIH) coincide with agricultural areas with annual irrigation (permanent and semi-permanent), as well as chaparral and secondary shrubby oak forest vegetation. Santa Cruz (SON) coincides with agricultural areas with annual irrigation (permanent and semi-permanent) and human settlements. For Mexicali (BC) it is associated with desert chaparral and chaparral.

Considering the main parameters associated with each study region, the maximum incidence of *Campylobacter* spp. in the Janos (CHIH) region was related to the presence of a pH ranging from 6 to 8 and salinity from 0.2 to 1.9, in contrast to Mexicali (BC) which can be very high and in Santa Cruz (SON) extremely low for both parameters. For Janos (CHIH), the fine soil texture, associated with high incidence, is more homogeneous throughout the region, while in Mexicali (BC) it is associated with small patches. In Santa Cruz (SON), despite a homogeneous composition of fine soil texture, the other characteristics associated with high risk of *Campylobacter* spp. incidence are lacking. In Janos (CHIH), soil moisture content is more variable and ranges from 2 to 8 months, while in Mexicali (BC) most of the soil is dry and in Santa Cruz (SON) soil moisture is present only 4 to 5 months per year.

With respect to temperature, if we compare the three municipalities, in Janos (CHIH) the average temperature (B1) is the highest and ranges from 20 to 27 °C, the maximum temperature of the warmest month (B5) from 35 to 37 °C, the average temperature of the rainiest month (B8) from 24 to 27 °C and the average temperature of the warmest quarter (B10) is from 21 to 22.3 °C. In the case of annual precipitation (B12) Janos (CHIH) falls within a range of 267 to 458.7 mm which is more variable compared to Mexicali (BC) which ranges from 30.6 to 271.1 mm and Santa Cruz (SON) which ranges from 450.2 to 509.5 mm.

• I do not feel that the Figure titles and legends give me enough information to understand the figures completely.

Figure corrected.

Line 201 References to ANUCLIM and Worldclim is needed here, particularly as the authors refer to them for the meaning of the colours in Figures 2-6, which are not given in a legend.

The bioclimatic parameters for the surface of the three municipalities of Northwest Mexico: Mexicali (BC), Santa Cruz (SON) and Janos (CHIH) were calculated by station from the 1981-2010 monthly meteorological database of the National Meteorological Service, following the ANUCLIM methodology also used in Worldclim (Fig. 2-6) [38, 39].

Corrected in the figures.

Line 214 Campylobacter spp., incidence

Corrected.

Discussion

• I struggle to understand how the authors put their findings into context with the literature. My expectations from the Introduction were that the model would describe the interplay of several factors to create ideal conditions for Campylobacter to be found but the authors seem to discuss individual factors such as humidity or temperature. I am obviously not an expert on this but I feel that more could have been made of the data?

Information included:

It is clear that any approach to investigating possible environmental factors associated with the increase in *Campylobacter* spp. cases must incorporate factors that may affect host exposure, including climatic conditions, soil types or conditions, as well as land use issues. Unlike other foodborne pathogens, *Campylobacter spp.* have generally been considered environmentally sensitive, fragile, and unable to multiply outside the host as a consequence of their thermophilic and microerophilic nature [16, 44, 45]. However, recent studies have shown it to be more resistant than previously thought [42] by reaching the viable but non-culturable cell (VBNC) stage and infecting susceptible hosts [46, 11]. Furthermore, the soil has been demonstrated to significantly contribute to the persistence of the bacterium in the environment. This is attributed to its relative significance as a potential infection source, linked to the bacterium's resistance to environmental conditions [18, 47]. With this study we intend to improve our understanding of the presence of *Campylobacter* spp. due to edaphic factors, presence of water bodies, vegetation and land use and the integration with meteorological factors that influence the distribution of *Campylobacter* spp. in the environment moderated by the combination of factors mentioned above.

We found that the two municipalities (Janos and Santa Cruz) with the highest risk zones for the presence of *Campylobacter* spp. agricultural soils with annual irrigation had a relationship

with high incidence of the bacterium. It has been shown that landscapes with a larger agricultural extension with permanent irrigation have a higher environmental contamination of *Campylobacter* spp. [48]. In addition, meteorological conditions in Janos (CHIH) provide higher soil moisture and less extreme temperatures compared to the other two municipalities. This may favor the presence and survival of *Campylobacter* spp. due to more stable temperatures and higher soil moisture [24]. Soil-borne pathogens survive better in a more open soil matrix that allows greater soil penetration and contains more organic matter, with less desiccation during dry weather. [49]. Although published data on *Campylobacter* spp. survival in northern Mexico are not available, it would be expected that survival would be lower in regions with greater desiccation. Additionally, runoff is generally lower in less compacted soils [Sanderson, 50], so the fine soil texture mostly found in Janos (CHIH) may favor *Campylobacter* spp. survival, favored by a pH and salinity suitable for their survival and therefore an increase in *Campylobacter* spp. exposure, especially those species known for their high survival in the environment such as *C. jejuni* [50].

• Overall, the lack of Campylobacter spp. data is mentioned several times, would a lack of data or differences in reporting between the areas skew the results of the model? If so, this needs to be discussed in more detail.

To resolve this lack of information, a systematic data search was carried out to establish the information required for the analysis, and clarified in the methodology:

A systematic search of world scientific literature was carried out in which edaphic, meteorological, water bodies, vegetation and land use factors associated with the presence of *Campylobacter* spp. in soil were established. At the same time, epidemiological records of *Campylobacter* spp. were consulted, without success, from Mexican public health and agricultural authorities and epidemiological bulletins. According to what was consulted in these institutions and corroborated by the systematic search carried out, it was not possible to obtain data for the northwestern region of Mexico that would be useful for our study.

• The conclusions section is not a conclusion but more a discussion of limitations, I would suggest discussing the limitations and expand on the conclusions described in the Abstract at the end of the discussion section.

Corrected:

The use of the kriging method of interpolation of unsampled areas is a tool that can help decision-makers to establish preventive and control strategies in high-risk areas and especially with infectious agents with complex cycles. Of the three municipalities evaluated, Janos (CHIH), has the highest number of geographic areas classified as high and medium incidence, followed by Santa Cruz (SON) and Mexicali (BC) with the highest number of geographic areas of low incidence zones.

In northern Mexico, known for its desert and extreme climatic conditions, the risk of the presence of *Campylobacter* spp. in the environment is mainly associated with agricultural areas with annual irrigation and low vegetation, regions that favor the survival and maintenance of *Campylobacter* spp. This, together with less extreme weather and soil conditions, mainly in the municipality of Janos (CHIH), favors humid environments with pH, salinity and soil texture conditions suitable for the maintenance of the bacteria.

Line 220 Campylobacter is a facultative anaerobe, so I think this needs rephrasing for accuracy.

Corrected.

It is clear that any approach to investigating possible environmental factors associated with the increase in *Campylobacter* spp. cases must incorporate factors that may affect host exposure, including climatic conditions, soil types or conditions, as well as land use issues. Unlike other foodborne pathogens, *Campylobacter spp.* have generally been considered environmentally sensitive, fragile, and unable to multiply outside the host as a consequence of their thermophilic and microerophilic nature [16, 44, 45]. However, recent studies have shown it to be more resistant than previously thought [42] by reaching the viable but non-culturable cell (VBNC) stage and infecting susceptible hosts [46, 11].

Line 222 the authors state that "recent studies have shown…" yet one of the papers referenced is from 2007

Corrected. Reference deleted.

Line 223 Viable but non culturable state

Corrected.

Line 224-226 This sentence is hard to understand, do the authors mean maintenance instead of permanence for example? Please rephrase.

Corrected.

Furthermore, the soil has been demonstrated to significantly contribute to the persistence of the bacterium in the environment. This is attributed to its relative significance as a potential infection source, linked to the bacterium's resistance to environmental conditions [18, 47].

Line 236 C. jejuni

Corrected.

Line 238 the municipality

Corrected.

Line 251 Rollins et al is the original reference for the 120 days, not the papers sited, other papers show a shorter duration so this should be acknowledged.

Corrected.

Survival in water varies among studies, for example Rollins et al. [51] reports a survival of 120 days, while Buswell et al. [52] reports a survival of 29 days. Furthermore, Nilson et al. [53] evaluated the variation of survival in water depending on seasonality, and in the case of *C. jejuni*, a significantly better survival was observed in autumn than in spring. [51, 52, 52].

Line 323/324 “Campylobacter spp. are the second most frequently reported cause of foodborne illness.” Where? In Mexico or globally? Also this is not the main conclusion of this data, see comment above.

Sentence deleted.
